# Generalist herbivore response to volatile chemical induction varies along a gradient in soil salinization

**DOI:** 10.1038/s41598-022-05764-0

**Published:** 2022-02-01

**Authors:** Jocelyn M. Marsack, Brian M. Connolly

**Affiliations:** 1 Department of Biology, Eastern Michigan University, 441 Mark Jefferson Science Complex, Ypsilanti, MI 48197 USA; 2 Biology Department, Gonzaga University, 502 E Boone Ave, Spokane, WA 99258 USA

**Keywords:** Ecology, Plant sciences, Ecology, Environmental sciences

## Abstract

Elevated soil salinity directly modifies plant physiology and indirectly alters the biotic interactions that shape plant performance. However, it is unclear how soil salinization interacts with plant defenses to alter patterns of leaf consumption or herbivore survival, development, and performance. In this study, we carried out laboratory feeding trials and a common garden experiment to investigate how gradients in soil salinization interact with plant induction status (modified via exogenous application of methyl jasmonate [MeJA]) to influence feeding consumption and performance of the generalist herbivore *Spodoptera exigua* on tomato (*Solanum lycoperscium*) plants. Our results showed that *S. exigua* consumed less leaf tissue from tomatoes treated with ≥ 50 mM NaCl; at these higher salinity treatments, these herbivores were less likely to pupate and died more quickly. Treatment with MeJA only reduced leaf consumption in the 0 mM NaCl treatment. Our common garden study demonstrated that natural populations of leaf chewing herbivores were less likely to damage tomatoes treated with > 50 mM NaCl solutions. Treatment with MeJA in the common garden reduced damage from natural populations of herbivores, but only for salt treatments at the 50 mM NaCl concentration level and we did observe variation in herbivore damage between cohorts in common garden trials. These results suggest that both soil salinization and volatile jasmonate signals may generate complementary shifts in decreased plant quality for herbivores. Overall, our study concludes that soil salinization could be a potential driver in spatial patterns of variation in both herbivory and herbivore demography.

## Introduction

Anthropogenic chemical inputs (e.g., CO_2_, O_3_, mineral salts) modify plant demography by influencing plant physiology^1^ and performance^2^. Further, they may also *indirectly* modify plant ecology by altering the biotic interactions structuring plant survival and performance^3^. Nitrogen deposition, for example, can increase herbivory^4^ and alters plant-mycorrhizae associations^5^. Elevated carbon dioxide alters herbivory^6^, but responses depend on temperature^7^ and concentrations of other chemicals (e.g., Ozone)^8^. Accurate descriptions of plant ecology require understanding of how anthropogenic chemicals modify the biotic interactions shaping plant demography.

Herbivory limits plant survival and growth^9^, and plants use mechanical and chemical defenses to deter herbivores^10^. Anthropogenic chemicals inputs can directly modify leaf quality for herbivores by altering limiting resources availability or by accumulating unpalatable compounds^6,11,12^. For example, plants damaged by herbivores release volatile organic compounds (VOCs) that signal upregulation of chemical defense in neighboring plants^10,13^. These VOCs can generate myriad and strong cascading effects on plant–herbivore interactions including, but not limited to, the attraction of predators and parasitoids that attack herbivores^14–16^ or increasing cannibalism rates among herbivores^17^. However, anthropogenic chemicals in plant communities can disrupt defensive chemistry by modifying signaling and/or receptor plant physiology or by diluting VOC concentrations, emulating VOC effect, or degrading the VOC signal into reaction products^18^. These shifts in VOC signaling create a potentially strong mismatch between a plant’s chemical defense profile and the intensity of local herbivory^19^. Our understanding of the effects of anthropogenic inputs can have on plant–herbivore interactions is developing, *see* Tao et al. (2013)^20^ and Forieri et al. (2016)^21^, but we still lack a clear understanding of how anthropogenic chemical inputs influence herbivory^21^ and modify induced defenses.

Sodium chloride (*hereafter* “salt”) is a common terrestrial mineral^22^ but human activities (e.g. road management, agricultural land use) have artificially elevated salt concentrations in agricultural and natural systems generating saline soils (i.e., soils with conductivity of saturation index > 4 dS m^−1^)^23^ with unclear effects on plant-animal interactions^24^. Concentrated soil salinities slow plant growth^25–27^ and delay flowering^28^ but have unclear effects on plant–herbivore interactions^24^. At high soil salinities, plants could increase salt concentrations in their tissues, decreasing plant quality, lowering leaf consumption, and limiting herbivore growth^29^. Alternatively, more salt within leaf tissue may stress plants resulting in greater herbivory (i.e., the environmental stress hypothesis)^30^. Finally, soil salinization may also modify chemical induction against herbivores by altering a plant’s physiology, its response to volatile hormones associated with herbivory (e.g., Methyl Jasmonante [MeJa]), or both. For instance, Dombrowski (2003)^31^ demonstrated that greater soil salinity upregulates proteinase inhibitors (i.e., enzymes that limit herbivore digestion and survival) in *Solanum lycopersicum* (tomato) seedlings, suggesting greater soil salinity may mimic the effect of defense signaling associated with herbivory or VOC signals from wounded neighboring plants, *see also* Forieri et al. (2016)^21^ and Younginger et al. (2009)^32^. However, it is still unclear how gradients in soil salinization interact with induction by volatile chemicals to modify leaf herbivory and generalist herbivore survival.

In our study, we used laboratory feeding trials and a common garden experiment to examine how gradients in soil salinization interact with plant induction status (modified via MeJA spray) to influence feeding consumption and performance of the generalist lepidopteran herbivore *Spodoptera exigua* on tomato (*Solanum lycoperscium*) plants. Laboratory trials isolated and quantified individual herbivore response to plants grown along a soil salinization gradient and these trials helped address potential mechanisms driving patterns of herbivory. Common garden trials measured the feeding response of natural populations of herbivores to plant induction status across a gradient of soil salinization treatments. We used tomato as study model species because this species generates consistent, reliable induction responses to environmental stressors (haline soils^31^; herbivore damage^13,15^) and previous work have demonstrated patterns of herbivory by generalist herbivores track closely with tomato induced status^17,33^. With this study, we aim to gain insight into how gradients in soil salinization affect patterns of leaf consumption and herbivore survival in the presence and absence of volatile plant hormones associated with imminent herbivory.

## Methods

### Laboratory feeding trial, herbivore performance and survival, and oxidative stress

To estimate the interactive effect of salt stress and chemical induction on generalist feeding on tomato, we conducted no-choice feeding trials with *S. exigua*. This herbivore is an agricultural pest on tomato, commonly used in feeding assays to determine how plant treatments (e.g., Methyl Jasmonate [MeJA] application, herbivore history, herbivore type, abiotic stress) influence feeding quality for a wide variety of plant^13,17,34,35^. Three cohorts of tomatoes (dates sown: 23 May 2018, 25 June 2018, and 2 July 2018) were grown and treated with aqueous sodium chloride solutions as described in Appendix 1; logistical constraints limited us to four discrete salt treatment levels (0, 50, 100, 150 mM NaCl). After twelve days of salt-treatment irrigation for each cohort, plants were randomly assigned to either receive an “induced” treatment (~ 12 mL spray with 1 mM MeJA in 2% EtOH with nanopure-filtered water) or “not induced” treatment (~ 12 mL spray with 2% EtOH with nanopure-filtered water). Treatment with 1.0 mM MeJA is able to elicit strong defense responses in tomatoes against *S. exigua* compared to more dilute concentrations (e.g., 0.1 mM MeJA), while minimizing the negative effects on plant growth rate observed at more concentrated MeJA treatments^17,33^. To limit cross-contamination, we separated plants by two meters within the laboratory and enclosed each plant within a 36.5 cm circular plastic shield (Clear Dura-Lar Film, Gratix, Maple Heights, Ohio, USA) with a lid. We treated plants one at a time and affixed lids on each enclosure before the next plant was treated. Plants remained in enclosures 20–30 min after the spray treatment before being returned to the greenhouse. There was a minimum of 12–15 plants of each salt-treatment level and MeJA-treatment level combination in each cohort, totaling no less than 36 plants (N = 36–45 plants) for each treatment combination across all cohorts.

Forty-eight hours after the induction treatment, we conducted no-choice feeding trials on single leaflets from the treated plants. We excised the fourth true leaflet of each treated plant and placed the leaflet on top of a damp germination blotter inside a plastic Petri dish (100 mm x 15 mm), the feeding arena. We then placed a single third-instar *S. exigua* larvae (provided by Benzon Research Inc. Carlisle, PA, USA) in each feeding arena and then sealed the dish with a strip of parafilm wax^33,35^. Sealing feeding arenas with wax prevents herbivores from escaping and minimizes desiccation of both herbivores and excised leaf tissue in feeding arenas. We recorded individual leaf and herbivore weights prior to each herbivory trial and measured these weights after 48 h of feeding. Previous work and pilot studies indicated this feeding duration was sufficient to capture differences in leaf damage while minimizing the likelihood of herbivore transitions between instars^33,35^.

To estimate the effect of leaf quality on herbivore survival and pupation, we returned herbivores to their feeding arenas after recording their 48-h weight; this experiment was only conducted for the second and third cohorts (defined above). We provided each herbivore with 2—4 g of leaf tissue from their assigned plant for the next 178 h; we provided sufficient leaf material so that each herbivore would not be food limited of the duration of the survival assay. Feeding arenas were sealed with parafilm as described above. At 48-h intervals, herbivore survival and development status (pupation vs. no pupation) were recorded. Due to logistical constraints, we subset this experiment to only evaluate: 1) S*. exigua* fate when fed on “induced” versus “not induced” tomato leaves when the control salt solution (0 mM) had been applied to the soil and 2) *S. exigua* fate when fed on “not induced” tomato leaves (i.e., sprayed with control solution) from each soil salinity treatment (four levels: 0 mM vs. 50 mM vs. 100 mM vs. 150 mM NaCl).

To explore how soil salinization and treatment with methyl jasmonate altered plant oxidative stress, we collected samples from the fifth true leaf of 40 different plants (20 MeJA-treated, 20 control sprayed) from the 25 June 2018 cohort. Leaf sampling occurred concurrently with lab herbivory trial setup. Leaves were harvested and then immediately stored at − 40 °C until total phenolics assay. We followed Ainsworth and Gillespie (2007)^36^ colorimetric total phenolic assay using Folin-Ciocalteu reagent and Gallic acid as a standard to estimate nanomoles of phenolics and other oxidative substrates (Gallic acid equivalents).

### Soil salinity under field conditions

We conducted a field experiment to evaluate how different soil salinities and induction via MeJA influenced herbivory by natural populations of herbivores. Two cohorts of *S. lycopersicum* (Cohort 1 sown: 25 June 2018; Cohort 2 sown: 2 July 2018) were grown in the greenhouse and treated with salt in an identical protocol to that described in Appendix 1. At the conclusion of the salt imposition treatment, we transplanted each cohort to common garden plots at Fish Lake Environmental Education Center (FLEEC) in Lapeer, Michigan (N 43° 6′ 41.776'' W 83° 14′ 58.455''). We transplanted plants in their original pots such that the level of soil media within each pot was flush with the surrounding soil surface; plants within plots were positioned ~ 25 cm apart. We kept plants within their pots to minimize soil contamination with salt while still permitting plants roots egress through holes on the bottom of the pot. Field cohort one was transplanted in the field 31 July 2018 and final herbivory and height measurements for field cohort one were taken 13 August 2018. Field cohort two was transplanted in the field 6 August 2018 and final herbivory and height measurements for field cohort two were taken 22 August 2018. Microclimate sensors (METER USA) installed ~ 30 m west of experimental plots recorded air temperature and precipitation at 15-min intervals during this field herbivory experiment. A summary of relative humidity and air temperature during these field trials is provided in Supplementary Files (Appendix 4); raw data associated with these and associated microclimate conditions in publicly available^37^.

Plants from each cohort were transplanted into common garden plots consisting of a 2 × 3 grid of 2 × 2 m plots (6 plots total); plots were separated by seven meters. We randomly assigned plants from each salt treatment level to a plot such that each plot contained at least one plant from each salt treatment level per cohort (Cohort 1, N = 24 total plants [3 plants per treatment combination]; Cohort 2, N = 32 total plants [4 plants per treatment combination]). We allowed plants to acclimate for 24 h and then all plants within randomly assigned plots were sprayed with either MeJA or a control spray treatment; three plots received the MeJA spray and three plots received the control spray. After two weeks, we measured: 1) plant height, 2) total leaflet number, 3) and the number of leaflets with > 10% leaf-chewing damage. We designated 10% damage as a cut-off for field-based herbivory damage based on: 1) previous field research suggesting > 10% leaf damage corresponds with severe and significant leaf-chewing damage done by insect herbivores^38^ and 2) proportion leaf tissue losses observed in control tomato plants in laboratory feeding trials (0.146 ± 0.047 proportion tissue removed ± SE; *see *"Results"). Our methods complied with relevant institutional, national, and international guidelines and regulations.

### Data analysis

We used a linear mixed model (LMM) to evaluate the influence of salt concentration and induction treatment on the proportion of leaf tissue consumed by the herbivore and herbivore mass change in our controlled lab feeding trial. We treated both salt concentration and induction treatment as discrete, fixed effects and cohort identity as a random effect in our models. All feeding arenas where the herbivore died or molted during the feeding trial were removed prior to analysis to ensure that we analyzed feeding trials where herbivores had similar opportunity to influence leaf mass. Additionally, we removed one outlier from leaf consumption analysis; this observation was greater than three standard deviations from the mean, was likely due to error in data entry, and does not markedly change results interpretation if included in analysis (See Appendix 2).

We used two separate Cox proportional hazard analyses to determine if 1) leaves from plants treated with methyl jasmonate influenced the rate of herbivore mortality, and 2) if leaves from plants treated with different salt solutions influenced the rate of herbivore mortality. We used a Pearson χ^2^ goodness of fit test to determine if the frequency of herbivores that pupated differed from expected distributions between NaCl treatment levels and MeJa treatment levels. We used general linear models to test how estimated total phenols and other oxidative substrates in leaf tissue related 1) between induction and salinity treatments, 2) to final plant height at harvest, 3) to the proportion leaf tissue consumed, and 4) to proportion herbivore mass change. Leaf tissue samples for phenol assays were only taken from one cohort; consequently, these linear models did not include random effects associated with cohort.

We used LMMs and generalized linear mixed models (GLMM, binomial distribution) to evaluate the influence of salt concentration, induction treatment, and cohort identity on plant height (LMM) and the extent of herbivore damage (i.e., the proportion of leaves on a plant with > 10% damage) (GLMM) in common garden plots. We included NaCl treatment nested with MeJa treatments as a random term to solve the split plot design. Cohort identity was included as a fixed effect to account for possible temporal differences in abiotic conditions and herbivore populations between the two cohorts. We treated salt concentration, induction treatment, and cohort identity as discrete, fixed effects. All analysis and graphics generation were conducted in R^39^ and we used the following R packages for data cleaning, result analysis, and graphics generation: “lme4”^40^ , “survival”^41^, “survminer”^42^, “dplyr”^43^ ,“emmeans”^44^, and “ggplot2”^45^.

## Results

### Laboratory feeding trial, herbivore performance and survival, and oxidative stress

**Figure 1:**
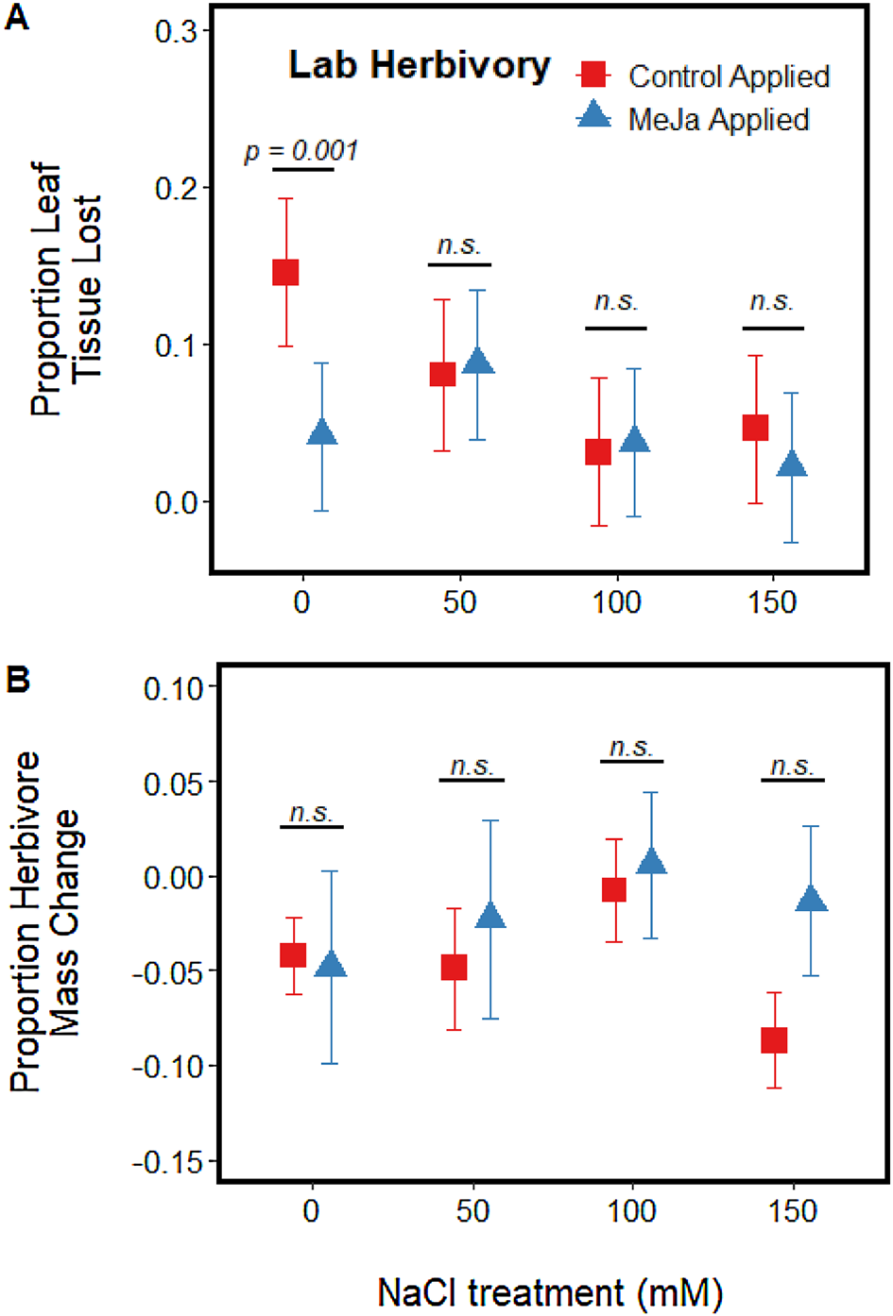
Effects of soil salinization at four concentrations of salt addition (0, 50 100, 150 mM NaCl) and plant induction via exogenous application of methyl jasmonate on (**A**) the proportion leaf tissue lost to generalist herbivore *Spodoptera exigua* and (**B**) the proportion change in herbivore mass during a 48 h no-choice laboratory feeding trial. Bars above each paired data points represent the results of a pairwise contrast testing the effect of chemical induction at each soil salinization level.

Our gradient of soil salinization concentrations influenced tomato performance and growth in patterns comparable to other similar research studies (*see* Appendix 1). In lab herbivory trials, leaves from tomato plants treated with 0 mM or 50 mM NaCl solution lost approximately three times more leaf tissue than leaves treated with greater concentration salt solutions (100 mM and 150 mM NaCl; Fig. 1A, Table 1). Chemical induction via methyl jasmonate reduced the amount of leaf tissue consumed by *S. exigua*, but only for plants that received the control salinization solution (Fig. 1A, Table 1). Proportion herbivore mass change were highly variable and did not differ between any salinization and induction treatment combination (Fig. 1B, Table 1; all pairwise contrast *p* values > 0.155).

**Table 1:** Results from linear mixed models testing for the effect of salt treatment in the soil, Methyl Jasmonate (MeJa) application and their interaction on feeding consumption (proportion of leaf consumed) and performance (proportion of herbivore mass change) of the generalist herbivore *Spodoptera exigua* on tomato (*Solanum lycoperscium*) plants in laboratory feeding trials.

Factor	F value	df	P value
***Response*: proportion leaf consumed**			
Salt treatment (NaCl)	3.96	3, 155	**0.009**
MeJa application (MJ)	3.59	1, 155	*0.060*
NaCl × MJ	2.76	3, 155	**0.044**
***Response*: proportion herbivore mass change**			
Salt treatment (NaCl)	0.81	3, 155	0.489
MeJa application (MJ)	1.13	1, 155	0.290
NaCl × MJ	0.44	3, 155	0.735

**Figure 2:**
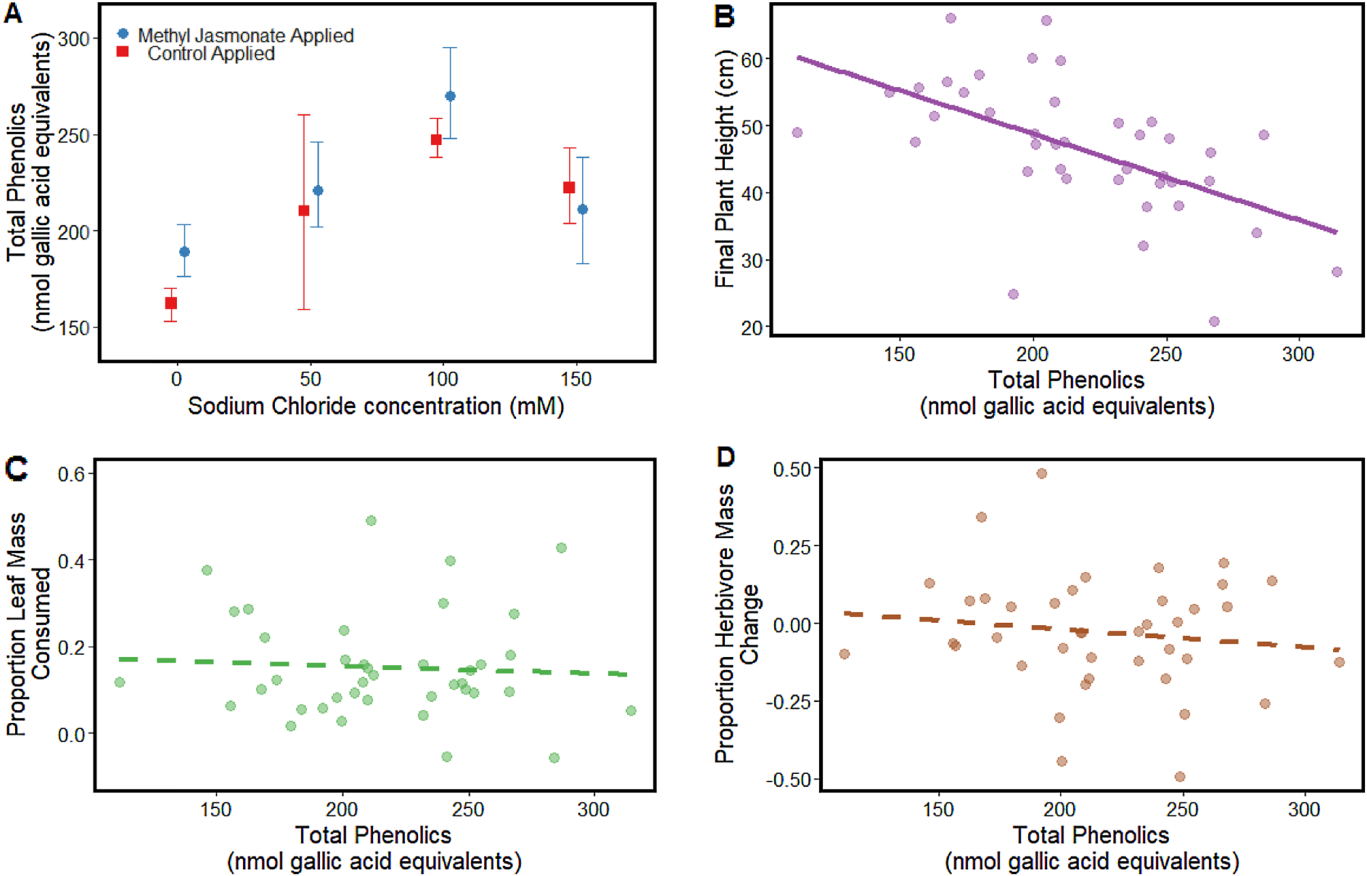
Effects of soil salinization at four concentrations of salt addition (0, 50 100, 150 mM NaCl) and plant induction via exogenous application of methyl jasmonate on (**A**) Folin-Ciocalteu reagent-based estimates of total oxidative substrates in leaf tissues. Correlation of total oxidative substrates in leaf tissue with (**B**) final plant height, (**C**) proportion leaf mass consumed, and (**D**) proportion herbivore mass change. Solid trend lines indicate statistically significant (Type I Error < 0.05) correlation between responses; dashed trend lines indicate no significant correlation between responses (Type I Error > 0.05).

Total leaf phenolics and other oxidative substrates were greater for plants treated with greater salt concentrations (Fig. 2A; F_3,32_ = 10.78, *p* < 0.001), but total leaf oxidative substrate content did not differ between leaves from the different MeJA treatments (F_1,32_ = 1.48, P = 0.232) nor did the effects of MeJA treatment on total leaf phenolics vary by NaCl treatment (NaCl treatment × MeJA induction treatment: F_3,32_ = 0.686, P = 0.567). Additionally, for the subset of lab herbivory samples for which we have leaf phenolics and oxidative substrates estimates, final plant height was significantly lower in plants with greater concentrations of total leaf phenolics (Fig. 2B; β = − 0.129, r_adj_ = 0.297, F_1,38_ = 17.48, p < 0.001). However, neither proportion leaf tissue consumed (Fig. 2C) nor proportion herbivore mass change (Fig. 2D) correlated with total phenolics and other oxidative substrates (all *p* values > 0.416).

**Figure 3:**
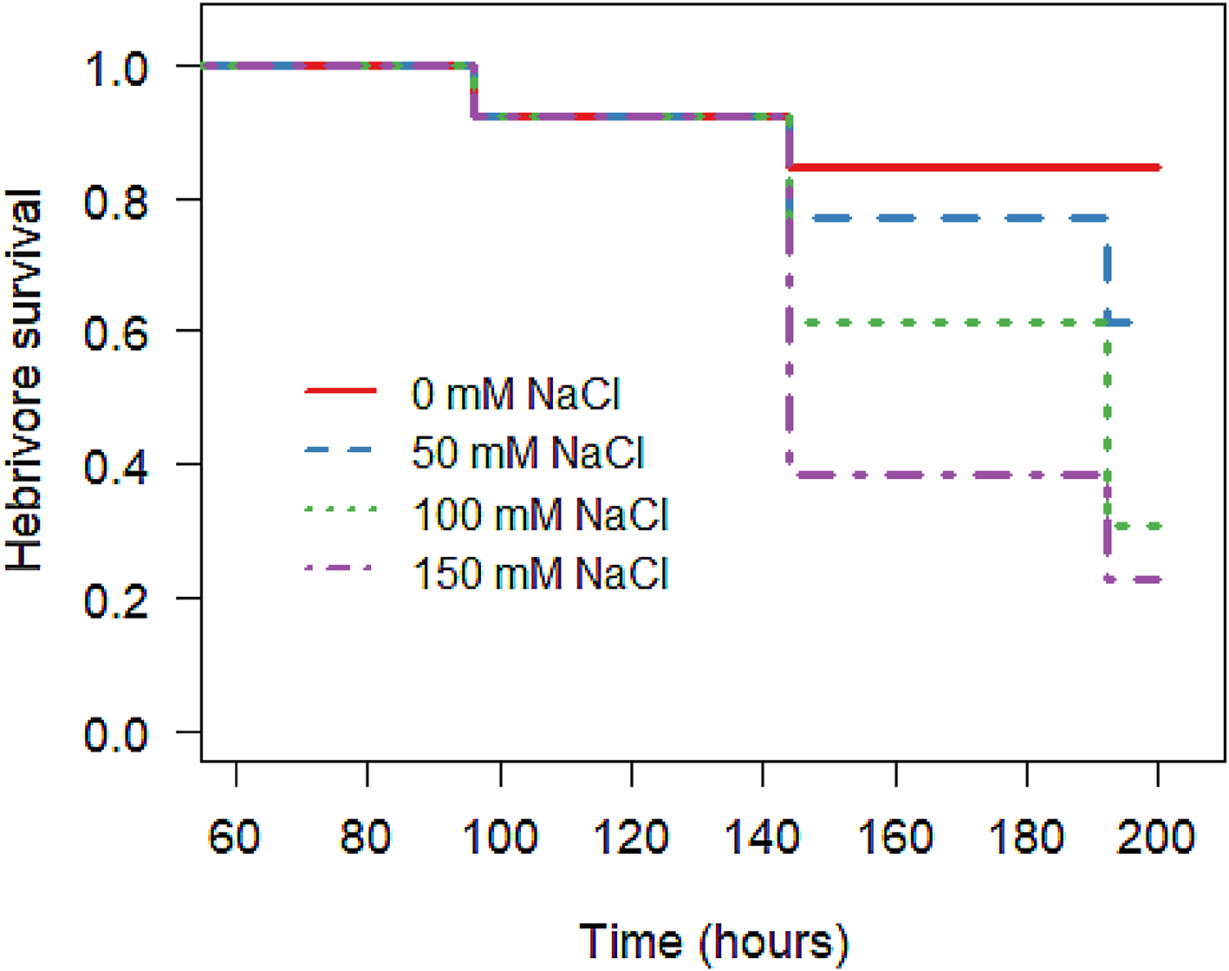
Effects of sodium chloride solution (0, 50 100, 150 mM NaCl) treated diet on *Spodoptera exigua* survival.

**Figure 4:**
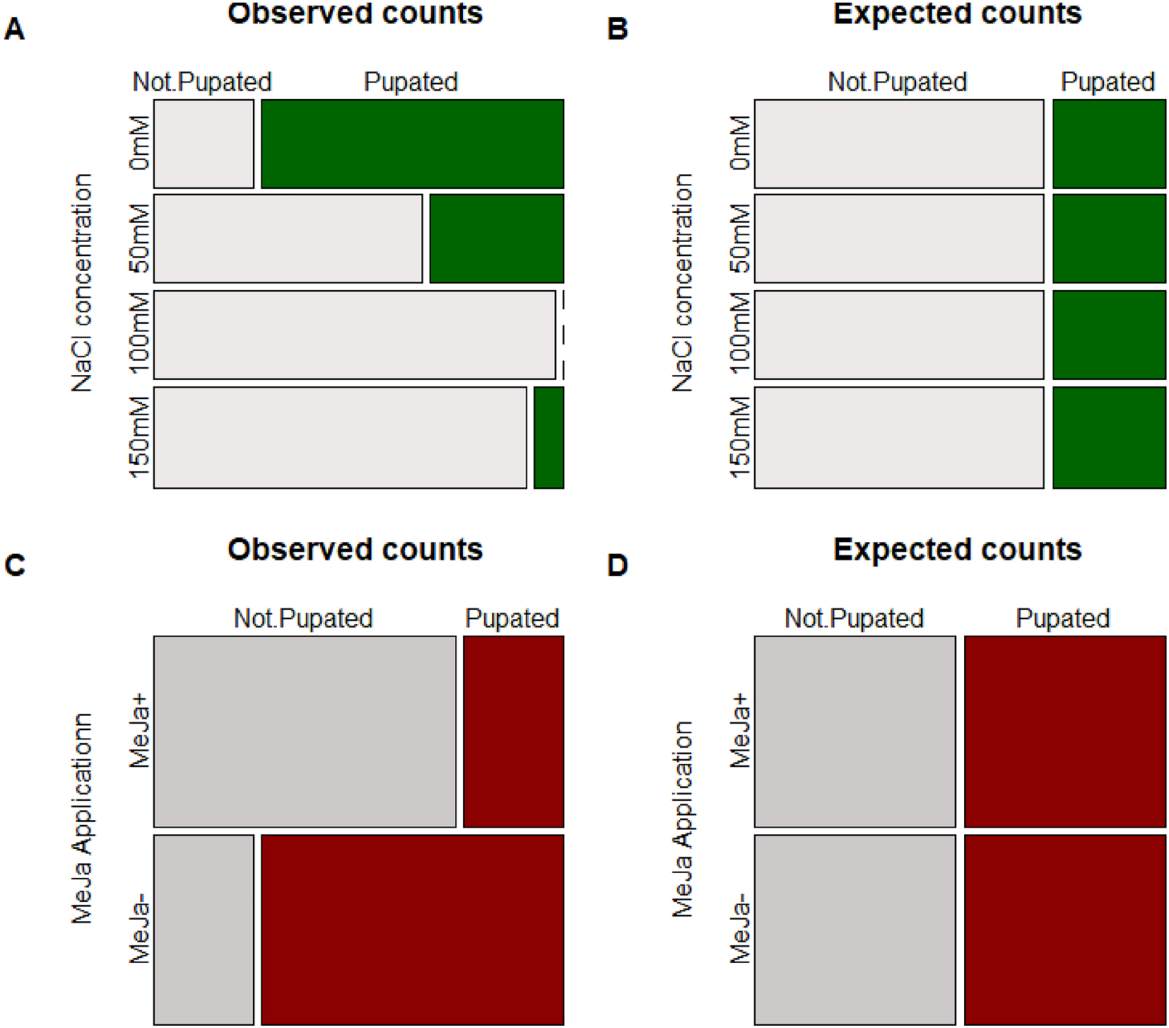
Effects of four different NaCl concentrations (0 mM, 50 mM, 100 mM, or 150 mM) on the (**A**) observed counts and (**B**) calculated expected frequencies of pupated *Spodoptera exigua* larva after 200 h on an assigned diet. Effects of plant induction via exogenous application of methyl jasmonate on the (**C**) observed counts and (**D**) calculated expected frequencies of pupated *Spodoptera exigua* larva after 200 h on an assigned diet.

Herbivores feed on diets of tomato leaves treated with 100 mM or 150 mM NaCl solution were almost twice as likely to die during the observation period (100 mM NaCl, Coef = 1.79, z = 2.28, *p* = 0.022; 150 mM NaCl, Coef = 2.10, z = 2.70, *p* = 0.007) than herbivores fed exclusively from the control treated plants (Fig. 3; χ^2^ = 12.31, d.f. = 3, *p* = 0.006). Herbivores fed for a prolonged period on a diet restricted to leaves from plants treated with methyl jasmonate tended to die more quickly than individuals feed on a diet of leaves from plants treated with the control solution (Figure S3; Coef = 1.38; χ^2^ = 3.59, d.f. = 1, *p* = 0.058). In these controlled feeding trials, the frequency of pupation deviated from expected equivalent distributions between soil salinization treatment levels (Fig. 4A, B; χ^2^ = 21.01, d.f. = 3, *p* < 0.001) and between MeJa treatment levels (Fig. 4C, D; χ^2^ = 4.17, d.f. = 1, *p* = 0.041).

### Soil salinity and induction under field conditions

**Figure 5:**
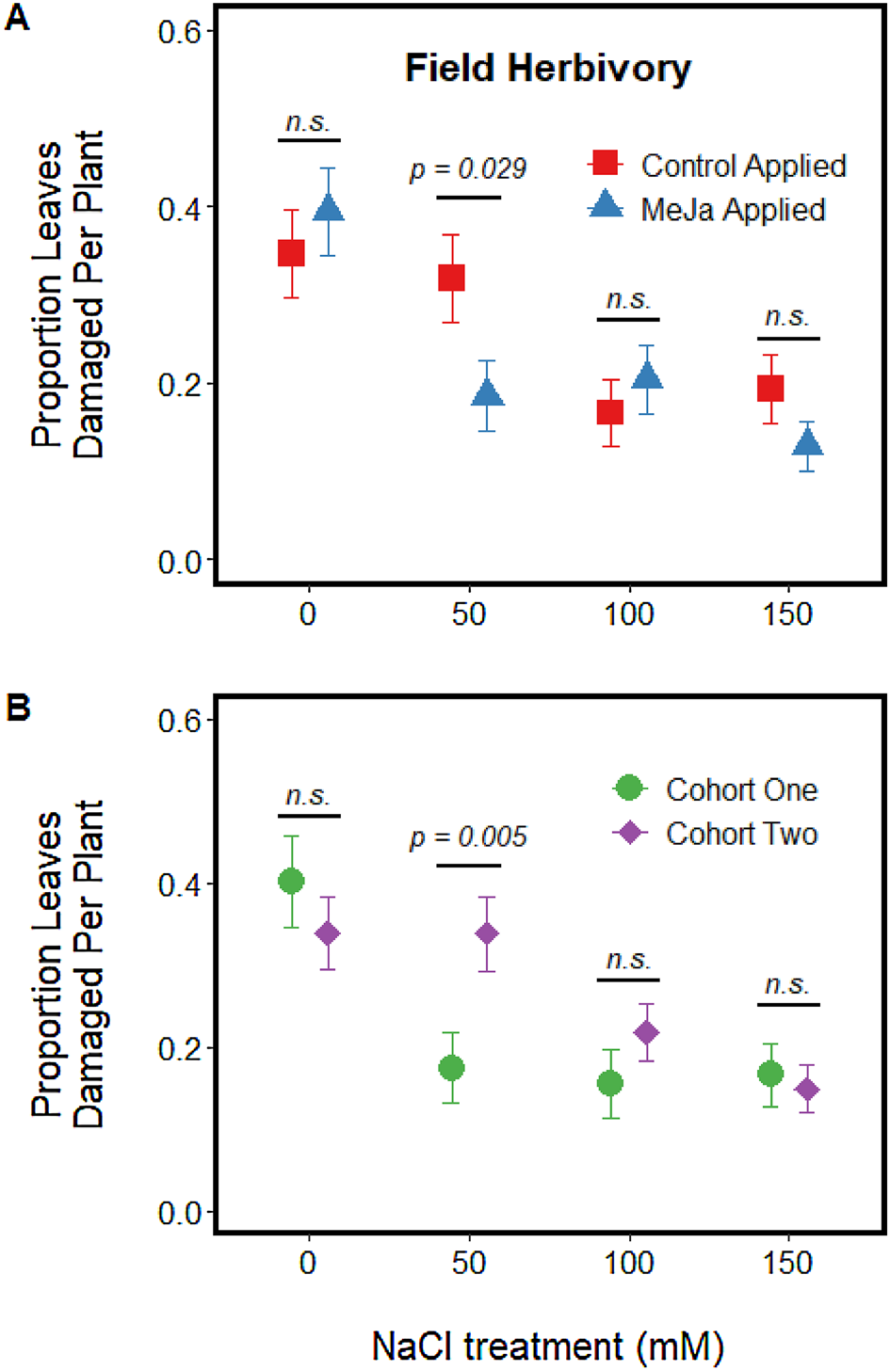
Effects of soil salinization at four concentrations of salt addition (0, 50 100, 150 mM NaCl) on the total proportion of leaves displaying > 10% herbivory in a common garden plot (**A**) after plant induction via exogenous application of methyl jasmonate and (**B**) between cohorts. Bars above each paired data points represent the results of a pairwise contrast testing the effect of chemical induction at each soil salinization level.

At the time of transplanting in the common garden experiment, total leaflet number did not differ between induction treatment levels (F_1,40.3_ = 0.07, *p* = 0.794) or soil salinization treatment levels (F_3,40.4_ = 1.96, *p* = 0.136). Overall, plants from the 100 mM and 150 mM NaCl soil salinization treatments displayed one-third to one-half the overall damage of the 0 mM and 50 mM NaCl soil treatment (Table 2). The effect of methyl jasmonate application on the total proportion of leaves that displayed damage varied by soil salinization treatment (Table 2), but chemical induction only significantly lowered the proportion damage at the 50 mM NaCl addition treatment (Fig. 5A; odds ratio = 2.205, *z-*ratio = 2.57, *p* = 0.010). Herbivore damage differed between cohorts at the 50 mM NaCl treatment level (Table 2); estimates of leaf damage at the 50 mM soil treatment for field cohort one was approximately half of the damage observed of plant from the 50 mM soil salinization treatment from field cohort two (Fig. 5B; odds ratio = 0.411, *z-*ratio = − 2.80, *p* = 0.005). Leaf damage did not differ between the cohorts at any other soil salinization treatment level (all *p* values > 0.217). Final field plant height differed by soil salinization treatment (Table S2, Fig. S4B), with control (0 mM salt treated plants) trending 10% taller than plants treated with 50 mM salt (*t* = 2.42, d.f. = 31.6, *p* = 0.094), 22% taller than plants treated with 100 mM salt (*t* = 4.92, d.f. = 31.4, *p* < 0.001), and 16% taller than plants treated with 150 mM salt (*t* = 3.88, d.f. = 31.4, *p* = 0.003). Final field height did not differ significantly for any other pairwise comparison (all *p* values > 0.103).

**Table 2:** Results from linear mixed models testing for the effect of salt treatment in the soil, Methyl Jasmonate (MeJa) application, cohort identification and their interactions on feeding consumption by the generalist herbivore *Spodoptera exigua* on tomato (*Solanum lycoperscium*) plants after two weeks in a field common garden.

Factor	χ^2^ value	df	P value
**Insect damage on field plants at two week census**			
Salt Treatment (NaCl)	46.68	3	** < 0.001**
MeJA application (MJ)	1.23	1	0.268
Cohort Identification (CH)	0.85	1	0.357
NaCl × MJ	10.20	3	**0.017**
NaCl × CH	9.87	3	**0.020**
MJ × CH	0.36	1	0.547
NaCl × MJ × CH	2.44	3	0.487

## Discussion

Our results showed that soil salinization had direct negative effects on plant performance (Fig. S1; *see* Katerji et al. 2003)^46^, limited herbivory^24^ (Figs. 1, 5) and modified herbivore development and survival^47^ (Figs. 3, 4). Our work demonstrated that plants grown in soils regularly exposed to concentrated salt solutions (e.g., ≥ 50 mM NaCl) experience less leaf consumption by generalist herbivores and the survival of these herbivores is likely limited by the reduction in leaf tissue quality. Our work also demonstrated that induction via methyl jasmonate spray—while reducing leaf consumption at concentrations of NaCl application ≤ 50 mM—has little effect on herbivore damage at greater concentrations of NaCl application.

Thresholds in salt concentrations within the soil generate direct negative effects on plant performance and growth. Tomato growth, for example, is notably susceptible to the effects of soil salinization. Katerji et al. (2003)^46^ demonstrated that, similar to other common agricultural crops, increased soil salinization directly limits tomato yield and physiological performance (i.e., evapotranspiration, pre-dawn leaf water potential, stomatal resistance). Our work corroborates this research demonstrating that, with regular applications of solutions > 50 mM sodium chloride, tomato accumulates compounds associated with oxidative stress (i.e., phenolics and other oxidative substrates, Fig. 2A) and exhibits slower physiological processes and growth (Fig. S1). Importantly, growth and stomatal conductance between our 0 mM and 50 mM sodium chloride did not differ, suggesting an important physiological tolerance threshold for tomato is surpassed when solutions > 50 mM sodium chloride are regularly applied. More experimentation using smaller concentration range increments between 0 and 50 mM sodium chloride will be necessary to parameterize tomato physiological response to soil salinization, particularly if these physiological responses to salt addition are non-linear over this concentration range or tomato varieties differ in their tolerance to soil salinity^48^.

Soil salinization and methyl jasmonate applications significantly reduced tomato tissue quality for *S. exigua* with lower larva mass gain and greater larval mortality on salt-treated plants in no-choice feeding trials. Poor leaf tissue quality on host plants generates unique behavioral responses in resident insect herbivores^24,49^. Poor leaf quality may increase larval lepidopteran dispersal^50,51^, resulting in significantly increased predation risk on these herbivores as the larva migrate between host plants^51^. Poor leaf tissue quality can also stimulate intraguild aggression resulting in increased rates of cannibalism^17^. Loss of generalist herbivores to cannibalism can generate growth benefits for host plants^17^ and may increase herbivore mortality by increasing viral load transmission between herbivores^52^- *but see* Elderd (2019)^53^ -but whether such benefits would persist for salt-stressed plants remains untested. Importantly, recent work indicates that several species of adult female lepidopterans oviposit similarly between plants enriched with sodium chloride and plants untreated with sodium chloride^54^. As sodium-enriched plants are poor food for larval lepidopterans (e.g., Fig. 3) and may delay or preclude pupation (Fig. 4A), soil salinization may directly affect lepidopteran demography. Reduced larval survival due to sodium-enriched, poor-quality host plants may be of concern particularly for endangered lepidopterans (e.g., the monarch butterfly, *Danaus plexippus*)^54^ surviving in or migrating through fragmented or urbanized habitats that contain artificially elevated soil salt concentrations. Plants in these salinization-prone habitats, however, may differ in salt tolerance^55^ suggesting the impacts of salinization on lepidopteran survival and performance may be a function of lepidopteran specialization and species-specific plant tolerance to soil salinization.

Herbivory in the field and in the lab followed two similar patterns: herbivory declined at more concentrated soil salinization treatments and treatment with MeJa typically deterred herbivory but only at lower soil salinization concentrations. However, application of MeJa influenced leaf consumption only at 0 mM NaCl treatments under laboratory conditions (Fig. 1), but MeJa diminished herbivory for only 50 mM NaCl treated plants under field conditions (Fig. 5). Although it is unclear what generated these differences in MeJa response between soil salinization treatments, it is possible that differences in experimental time scale generated the observed trends. Field plants grew for two weeks after treatment with MeJa and salt imposition treatments were ceased following transplanting. The effects of MeJa treatment are often transient, with peak chemical induction for unstressed plants returning closer to constitutive levels 24–48 h after induction^56^; our laboratory feeding trial captured herbivory patterns during this window of peak induction response. Herbivory on field plants treated with 0 mM NaCl may be comparable between MeJa treatment levels because the extent of defensive chemistry, while initially differing between MeJa treatment levels, was largely comparable between the two MeJa treatment levels for the duration of the time these plants were in the field. Tomatoes in the field treated with 50 mM NaCl, however, had the joint stressors of soil salinization and treatment with MeJa. As soil salinization treatments can generate chemical induction like that observed following leaf wounding^31^, it is possible that the joint treatment of 50 mM NaCl and MeJa spray generated additive levels of defensive chemistry that drove patterns of herbivory that persisted for the two weeks these plants were in the field. Field studies examining temporal trends in secondary chemistry for MeJa-treated plants at different soil salinization levels would help inform if our observed trends are due to temporal differences in plant defensive chemistry.

Estimates of phenolics and other oxidative substrates using Folin-Ciocalteu reagent is a general assay that has been used previously to correlate herbivory, herbivore performance, and plant stress^57^. Importantly, this is a general assay^36^ and, while it effectively demonstrates how oxidative stress correlates with plant growth (Fig. 2B), this test does not quantify phenolic compounds closely linked to herbivore performance or leaf consumption (e.g., Chlorogenic acid, flavonoids, tannins)^58^. Unique insect herbivore guilds (i.e., leaf chewers, leaf miners, phloem feeders) respond differently to sodium-treated plants^59^ and may respond differently to different groups of phenolics^57^. Our work only examined the effects of soil salinization and induction status on leaf chewers but examining how a wider diversity of insect herbivore functional groups respond to soil salinization and plant induction will help predict how insect herbivores and associated food webs are influenced by greater soil salinity^24^.

Spatial variation in soil salinization may generate unique patterns of inter-plant signaling and defensive chemistry against herbivore attacks. At greater soil salinization concentrations, application of methyl jasmonate—a plant-signaling hormone commonly associated with risk of herbivory^10,13^—failed to generate significant shifts in synthesis of oxidative substrates (Fig. 2A), leaf consumption under laboratory conditions (Fig. 1), or the total amount of significant herbivore damage on plants in a common garden (Fig. 3). Our results highlight that abiotic stresses and plant hormones associated with biotic stresses may not always work additively on leaf palatability to generalist consumers. Rather, in our study, both salt-stress and jasmonate signals generated complementary shifts in tissue quality for the generalist herbivore *S. exigua* at salt concentrations > 50 mM. Interestingly, salt-stressed plants display an altered profile of volatile cues emission (e.g., shifts in methanol and isoprenoids) that can regulate the physiological rates of neighboring plants and “prime” neighboring plants against imminent abiotic stresses^60^. Although our work indicates that soil salinization decreases leaf quality and makes plants unresponsive to subsequent jasmonate signaling, it is still unclear 1) how salts mediating intra-plant signaling would spatially modify herbivory or 2) if lower herbivory mediated by soil salinity offsets physiological costs of growing in saline soils. Such studies, however, would be useful for predicting both resident plant and herbivore fate in areas subject to prolonged soil salinization^60^.

Soil salinization requires creative remediation techniques to mitigate deleterious legacies in agroecosystems and roadside habitats. For example, supplementing roadside or agricultural soil with targeted chemical treatments (e.g., calcium or salicylic acid additions) may mitigate the effects of elevated soil salinization on plant survival and performance^61,62^. Arbuscular mycorrhizal fungi can play an important role facilitating plant response to threats from both herbivores^63^ and elevated soil salinization^64^, prioritizing strong plant-fungi mutualism as a land management strategy where salt concentrations in the soil are elevated may equip plants with sufficient physiological tools to tolerate adverse conditions. Currently, it is unclear if, or how, remediation translates to variable herbivore survival and growth, but our work demonstrates managing directly for the physiological performance of plants by minimizing the effects of soil salinization will likely have indirect, positive effects for associated herbivores.

## Supplementary Information

Supplementary Information.
