# Supplementary material for: Generalist herbivore response to volatile chemical induction varies along a gradient in soil salinization
Source: Sci Rep. 2022 Feb 1;12:1689. doi: 10.1038/s41598-022-05764-0 (PMC8807617; doi:10.1038/s41598-022-05764-0)
Supplement: Supplementary file 1 — Supplementary Information. [file 41598_2022_5764_MOESM1_ESM.docx]

**Supplemental Material - Supporting Methods and Results:** Generalist herbivore response to volatile chemical induction varies along a gradient in soil salinization.

Jocelyn Marsack*^1^ and Brian M. Connolly^1,2^

^1^ Department of Biology, Eastern Michigan University

441 Mark Jefferson Science Complex, Ypsilanti, MI 48197

^2^ Biology Department, Gonzaga University

502 E Boone Ave, Spokane WA 99258

*correspondence author

^1^ email: [jmarsac2@emich.edu](mailto:jmarsac2@emich.edu)

^2^ email: [connollyb@gonzaga.edu](mailto:connollyb@gonzaga.edu)

**Supplemental Information**

*Appendix 1.* Physiological and growth response in tomato to increasing soil salinization.

**Methods**

We started our experiment by establishing how our soil salinization gradient treatment influenced tomato performance. We sowed tomato seeds (*Solanum lycopersicum* v. Moneymaker; Baker Heirloom Seed Co., Missouri, USA; Thaler et al. 2010, Orrock et al. 2017, 2018) in individual 8.7 x 8.7 cm pots with standard potting media on 22 April 2018. We placed pots in greenhouse trays with holes to prevent cross-contamination by the salt treatment. Tray position on the bench was rotated every 3-5 days to control for possible differences in microclimate. We left the first seedling to emerge in each pot intact and we removed all subsequent emergents. Plants were irrigated regularly to maintain saturated soils. Tomatoes grew for 18 days in the greenhouse (23-28°C), after which we started our soil salinity treatment. One of five different NaCl solutions (0, 25, 50, 100, 150 mM NaCl [Product Number: S5886; Sigma-Aldrich, Missouri, USA] in nanopure-filtered water) was used to irrigate plants twice daily at 08:00 and 16:00 h (18 plants per NaCl solution, 90 plants total) for twelve days. Soil salinization levels reflect ranges previously used in previous work evaluating salinization stress in agricultural plants (e.g. Forieri et al. 2016) and tomato in particular (e.g., Dombrowski 2003, Zribi et al. 2009). We irrigated plants with their assigned salt solution until the solution drained freely from the pot.

We measured plant height at the start and conclusion of the salinization treatment. Twelve days after the initiation of soil salinization treatment, we measured stomatal conductance using a SC-1 Leaf Porometer (METER Group, Pullman, WA, USA) on the fourth-true leaf for a subset of plants (10 plants in 0, 50, 100, 150 mM NaCl treatments, 9 plants in the 25 mM NaCl treatment). Stomatal conductance measurements were made between 09:00 and 11:00 hours and we randomized the order of plant sampling across salt treatment levels. Plants were then destructively harvested, dried for 72 hours in a drying oven (65°C), and then weighed to measure biomass production.

*Data Analysis*

We calculated relative growth rate (RGR) for each plant based on the change height observed over the salt imposition period; tomato height correlates well with dry aboveground biomass (Fig. S1A, Fig. S5A; r^2^ = 0.484, F_1,88_ = 82.61, *p* < 0.001). We used a general linear model to evaluate the influence of salt concentration (as a continuous fixed factor) on tomato aboveground biomass, final plant height, RGR, and stomatal conductance. Final plant height was squared, stomatal conductance measures were square root transformed, and final biomass and RGR were log transformed prior to analysis to ensure that models met assumptions of normal error distribution (Crawley 2013).

**Results**

In laboratory growing trials, final dry aboveground biomass did not differ between soil salinization treatments (Fig. S1B; F_1,88_ = 0.04, *p* = 0.834), but final plant height tended to decrease at greater soil salinization concentrations (Fig. S1C, β = -4.86, r^2^ = 0.027, F_1,88_ = 3.50, *p* = 0.065; *see also* Fig. S5A). The relative growth rate of tomato treated with ≤50 mM NaCl treatment were almost double that of plants treated with greater salt concentrations (Fig. S1D; β = -0.002, r^2^ = 0.355, F_1,88_ = 48.53, *p* < 0.001) and stomatal conductance was significantly faster for tomato plants treated with ≤50 mM NaCl treatment compared to plants treated with greater salt concentrations (Fig. S1E; β = -0.053, r^2^ = 0.574, F_1,47_ = 63.2, *p* < 0.001).


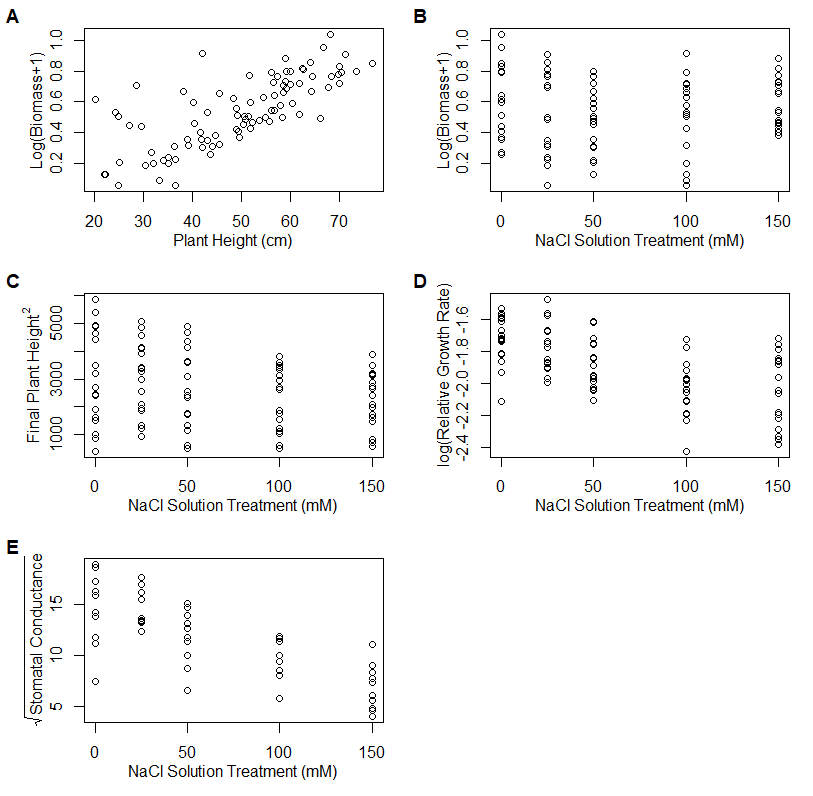


**Figure S1**. Summary of tomato growth as it corresponds to the relationships between tomato plant height and dry above ground biomass (**A**). We also report figures demonstrating how 12 days of soil treatment with each of five NaCl solution treatments (0mM, 25mM, 50mM, 100mM, and 150mM) influence final plant biomass (**B**), final plant height (**C**), relative growth rate (**D**, based on change in height), and stomatal conductance rates (**E**).

*Appendix 2.* Summary of lab herbivory experiment results with and without the outlier

Table S1. Results table for proportion leaf tissue consumed in laboratory feeding trials both including and excluding a single outlier.

| Factor | F - value | d.f. | P-value |
| --- | --- | --- | --- |
| *Response*: Proportion Leaf Consumed (outlier included) | | | |
| Salt Treatment (NaCl) | 2.46 | 3, 156 | *0.065* |
| MeJa application (MJ) | 4.61 | 1, 156 | **0.033** |
| NaCl × MJ | 3.60 | 3, 156 | **0.015** |
| *Response*: Proportion Leaf Consumed (outlier removed) | | | |
| Salt Treatment (NaCl) | 3.96 | 3, 155 | **0.009** |
| MeJa application (MJ) | 3.59 | 1, 155 | *0.060* |
| NaCl × MJ | 2.76 | 3, 155 | **0.044** |

*Appendix 3.* Summary of results from common garden experiment

Table S2. Results table for common garden plant height after two weeks under field conditions. Degrees of freedom calculation follow Kenward-Roger approximation.

| Factor | F value | d.f. | P-value |
| --- | --- | --- | --- |
| *Height of field plants at two week census* | | | |
| Salt Treatment (NaCl) | 8.77 | 3, 31.4 | **<0.001** |
| MeJA application (MJ) | 1.79 | 1, 3.5 | 0.261 |
| Cohort Identification (CH) | 1.47 | 1, 33.0 | 0.257 |
| NaCl × MJ | 0.98 | 3, 31.6 | 0.441 |
| NaCl × CH | 0.99 | 3, 31.8 | 0.421 |
| MJ × CH | 0.26 | 1, 33.1 | 0.650 |
| NaCl × MJ × CH | 2.74 | 3, 32.0 | *0.059* |

*Appendix 4.* Summary of microclimate conditions between cohorts


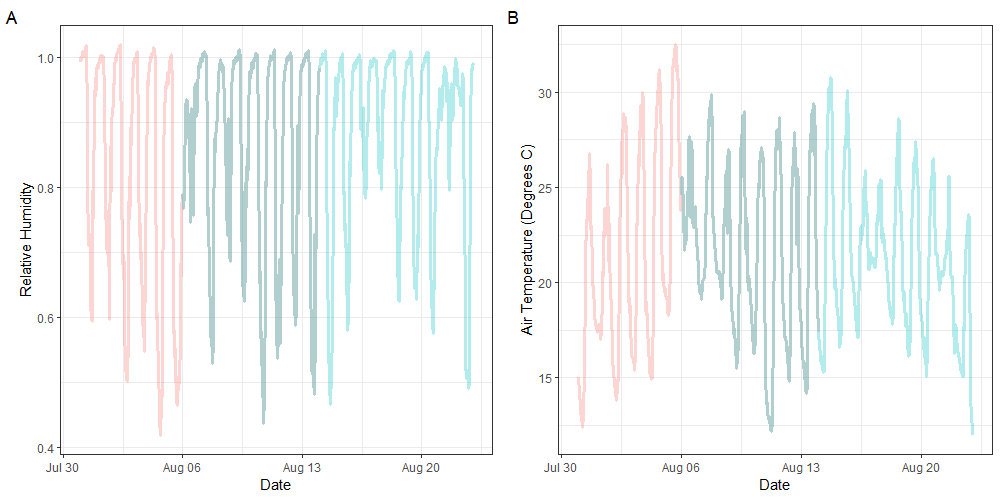


Figure S2. Visual summary of Relative Humidity (Panel A) and Air Temperature (Panel B) over the duration of field trails. Microclimate measurements follow the red line for cohort one, measures follow the light blue line for cohort two. Cohorts overlapped by one week and shared microclimate conditions from 6 August 2018 to 13 August 2018; this shared period is centered on each plot and follow the dark grey blue line.

Table S3. Table summarizing microclimatic conditions between the two field cohorts

| **Measurement** | **Cohort 1** | **Cohort 2** |
| --- | --- | --- |
| *Relative Humidity* |  |  |
| Mean ± Standard Deviation | 0.84 ± 0.17 | 0.87 ± 0.15 |
| Maximum | 1.02 | 1.01 |
| Minimum | 0.42 | 0.44 |
|  |  |  |
| *Temperature* (℃) |  |  |
| Mean ± Standard Deviation | 21.72 ± 4.88 | 21.43 ± 4.15 |
| Maximum | 32.5 | 30.8 |
| Minimum | 12.2 | 12.2 |

*Appendix 5*: Effects of induced leaf tissue diet on herbivore survival rate.


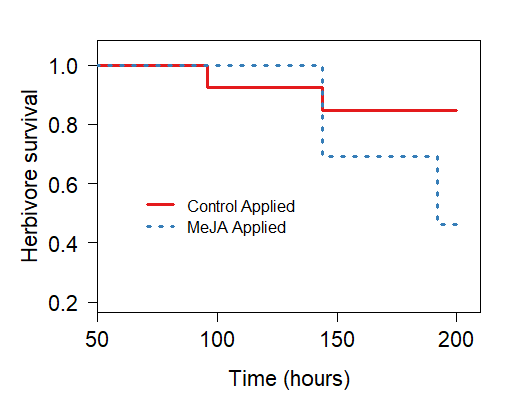


Figure S3. Survival curve for *Spodoptera exigua* fed on a diet consisting of tomato leaves from a plant treated with methyl jasmonate or with leaves from plants treated with a control spray.

*Appendix 6.* Effects of methyl jasmonate application on tomato growth at four different soil salinization treatment levels.


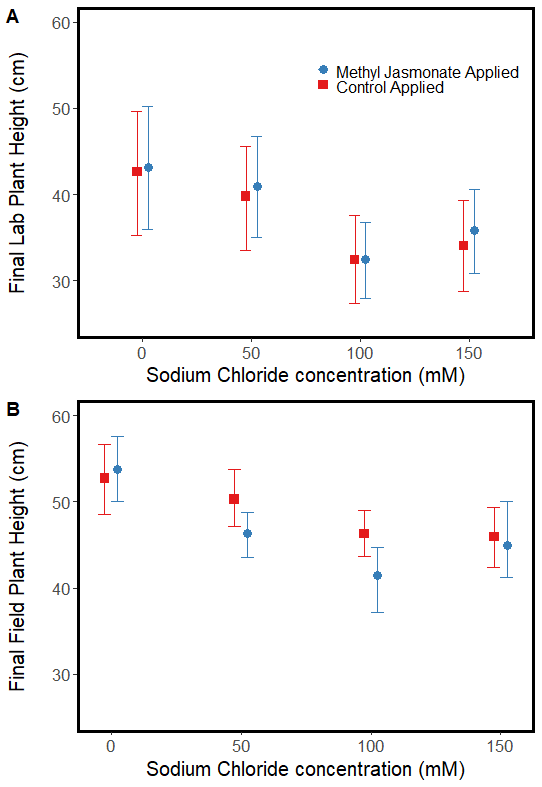


Figure S4. Estimates of the effects of methyl jasmonate spray (compared to control spray) on tomato growth (height) between tomatoes treated with four different sodium chloride concentrations of solutions and grown under (A) greenhouse conditions and (B) after two weeks under field conditions.
